# Growth in VLBW infants fed predominantly fortified maternal and donor human milk diets: a retrospective cohort study

**DOI:** 10.1186/1471-2431-12-124

**Published:** 2012-08-17

**Authors:** Tarah T Colaizy, Susan Carlson, Audrey F Saftlas, Frank H Morriss Jr

**Affiliations:** 1 Department of Pediatrics, Carver College of Medicine, University of Iowa, Iowa City, USA; 2 Department of Food and Nutrition, University of Iowa Hospitals and Clinics, Iowa City, USA; 3 Department of Epidemiology College of Public Health, University of Iowa, Iowa City, USA

## Abstract

**Background:**

To determine the effect of human milk, maternal and donor, on in-hospital growth of very low birthweight (VLBW) infants. We performed a retrospective cohort study comparing in-hospital growth in VLBW infants by proportion of human milk diet, including subgroup analysis by maternal or donor milk type. Primary outcome was change in weight z-score from birth to hospital discharge.

**Methods:**

Retrospective cohort study.

**Results:**

171 infants with median gestational age 27 weeks (IQR 25.4, 28.9) and median birthweight 899 g (IQR 724, 1064) were included. 97% of infants received human milk, 51% received > 75% of all enteral intake as human milk. 16% of infants were small-for-gestational age (SGA, < 10^th^ percentile) at birth, and 34% of infants were SGA at discharge. Infants fed >75% human milk had a greater negative change in weight z-score from birth to discharge compared to infants receiving < 75% (−0.6 vs, -0.4, p = 0.03). Protein and caloric supplementation beyond standard human milk fortifier was related to human milk intake (p = 0.04). Among infants receiving > 75% human milk, there was no significant difference in change in weight z-score by milk type (donor −0.84, maternal −0.56, mixed −0.45, p = 0.54). Infants receiving >75% donor milk had higher rates of SGA status at discharge than those fed maternal or mixed milk (56% vs. 35% (maternal), 21% (mixed), p = 0.08).

**Conclusions:**

VLBW infants can grow appropriately when fed predominantly fortified human milk. However, VLBW infants fed >75% human milk are at greater risk of poor growth than those fed less human milk. This risk may be highest in those fed predominantly donor human milk.

## Background

Maternal milk diets have been associated with advantages for extremely low birthweight (ELBW) infants. ELBW infants fed maternal milk have lower rates of necrotizing enterocolitis (NEC) [1-3], the combined outcome of NEC or death [4], late onset sepsis [2,5,6], and have superior neurodevelopmental outcomes compared with those fed preterm formulas [7,8].

However, maternal milk diets have also been associated with inferior in-hospital growth when compared with preterm formula. Studies performed prior to the current practice of human milk fortification demonstrated poorer growth in maternal–milk-fed infants compared with those fed formula [9], although neurodevelopmental advantages were associated with human milk intake [7,10]. Results of subsequent studies of maternal milk intake and growth in VLBW infants during the era of routine fortification have been mixed [1,2,11]. Slower in-hospital growth associated with a maternal milk diet in ELBW infants remains a concern, because poor in-hospital growth in ELBW infants is common [12-14] and is associated with poor neurodevelopmental outcomes at 18–22 mo [15].

The relationship between human milk intake and growth becomes even less clear when donor human milk diets for ELBW infants are considered. Pooled, pasteurized donor human milk is a dietary intervention that is increasing in usage in the very low birthweight (VLBW) population in the US [16], but has not been well studied.. Early studies showed growth deficits with unfortified donor milk compared with formula and maternal milk [17,18]. A later study comparing fortified donor milk and preterm formula as supplements to fortified maternal milk also found slower growth associated with donor milk [1]. Studies of the impact of donor human milk diets on growth, particularly diets composed predominantly of fortified donor human milk, are needed to determine the safety and efficacy of this intervention. Further studies of fortified maternal milk diets are also needed to determine the growth effects in the era of routine fortification. For the purposes of this study, ‘maternal milk’ refers to human milk produced by the mother of an infant. In contrast, ‘donor human milk’ refers to milk expressed by volunteer milk donors, processed by the Mother’s Milk Bank of Iowa.

We undertook a retrospective cohort study to assess in-hospital growth, as defined by change in weight z-score for gestational age between birth and hospital discharge, and incidence of growth failure defined as < 10^th^ percentile weight for corrected gestational age at hospital discharge among infants weighing ≤ 1250 g at birth fed predominantly human milk diets.

Our objective was to study in-hospital growth of a subset of VLBW infants treated in a center with extensive human milk usage, including extensive use of donor milk. We planned to study the effects of increasing proportions of human milk fed during hospitalization, as well as differential effects of predominantly maternal milk vs. predominantly donor human milk diets. We hypothesized that increasing fortified human milk intake would be associated with slower, but adequate, growth, and that growth would be slowest in infants fed fortified donor human milk.

## Methods

### Study population

We identified all infants born at University of Iowa Children’s Hospital NICU or admitted by transport within 24 hours of birth with birthweights ≤ 1250 g between January 1, 2003 and June 30, 2005 using a nutritional database maintained by a neonatal dietician. Although the formal definition of VLBW includes infants up to 1500 g at birth, our nutritional database includes only infants weighing ≤ 1250 g at birth. In this manuscript, all references to our study population as VLBW should be understood to refer **only** to the subset of VLBW infants we studied. We included for analysis all of those who were free of major chromosomal or structural congenital anomalies and were discharged home or transferred to another facility at ≥36 weeks’ gestational age. We excluded those who died during hospitalization or were transferred prior to 36 weeks to focus on growth in infants from birth through typical hospital discharge range. Clinical and demographic variables were collected retrospectively from the subjects’ medical records. The Mother’s Milk Bank of Iowa, a Human Milk Banking Association of North America (HMBNA) member bank, dispensed its first milk to the UICH NICU in May 2003. All donor human milk fed to infants in this study was obtained from the MMBI. Milk donors were unpaid volunteers, most of whom had delivered term infants. Milk from 3–10 donors was pooled and pasteurized by the Holder method in batches of 4.3 L in accordance with HMBANA standards. The MMBI does accept donations of milk from women who deliver preterm infants but does not process this milk separately or label milk as ‘preterm’ or ‘mature’. This study was approved by the University of Iowa IRB.

### Clinical variables

Intraventricular hemorrhage (IVH) was defined by highest grade recorded, retinopathy of prematurity (ROP) was defined by highest stage reported, necrotizing enterocolitis (NEC) was defined by Bell’s stage II or higher, late onset sepsis (LOS) was defined by a positive blood culture after 3 days of age accompanied by IV antibiotic therapy, and patent ductus arteriosus (PDA) was defined by presence of hemodynamically significant patent ductus on echocardiogram that was treated with indomethacin or surgical ligation. We also collected discharge home on supplemental oxygen.

### Nutritional intake and growth variables

Human milk intake data were collected from nursing flowsheets by recording separately daily intake of maternal and donor milk. Human milk intake was categorized by percent of total enteral intake during hospitalization (<25% of diet, 25-50% of diet, 51-75% of diet, and >75% of diet), combining donor and maternal milk intakes. All enteral intake that was not human milk was various infant formula preparations. The subgroup of infants receiving >75% of enteral intake as human milk were further subdivided for analysis by milk type: >75% donor milk, >75% maternal milk, and a >75% human milk diet of mixed donor and maternal milk. Postmenstrual age-specific z-scores, based on the Fenton growth chart, were calculated for birth weight, discharge weight, and discharge occipito-frontal circumference (OFC) [19]. SGA status at discharge was defined as < 10^th^ percentile for postmenstrual age according to the Fenton growth chart. We chose to use z-scores rather than weight measurements because this allows us to use hospital discharge as the outcome time point, which varies from subject to subject. It also provides standardization to a recognized scale, the widely-used and validated Fenton growth chart. If maintenance of the in-utero growth rate for VLBW infants is achieved, an infant born with a weight z-score of 0 (50^th^ percentile for postmenstrual age) will have the same weight z-score for postmenstrual age at discharge. Negative change in weight z-score from birth to discharge represents growth less than the predicted growth rate, whereas positive change represents a growth rate greater than the predicted growth rate.

### Study outcomes

The primary outcome of the study was change in weight z-score for postmenstrual age from birth through discharge, stratified by quantity of human milk received. Secondary outcomes included weight z-score for postmenstrual age at discharge and discharge SGA status by human milk intake. Subgroup analyses of change in weight z-score, discharge weight z-score, discharge OFC z-score, and discharge SGA status were performed on the infants receiving >75% human milk. Protein and calorie supplementation beyond 24 kcal/oz (2–2.3 g protein/dl) was also compared by human milk intake across the whole study population, and also within the >75% human milk subgroup.

### Nutritional management

Nutritional management of this subgroup of VLBW infants at UICH under daily guidance by the neonatal dietician was uniform during this study period, except for the introduction of donor human milk, which occurred 6 months into the 30 month study period. All infants received IV glucose within an hour of birth. Parenteral nutrition (TPN) with provision of 2–3 g/kg/day of protein and 1–2 g/kg/day of fat as Intralipid 20% (Baxter) was started on day of life 0 or 1. Parenteral protein administration goal was 3 g/kg/d, and lipid dose goal was 2 g/kg/d. Parenteral amino acid intakes were maintained at 3 g/kg/d until parenteral volume fell below 60 ml/kg/d and then maintained at 5 g/dl until TPN was discontinued. Glucose infusion rates were titrated to maintain blood glucose measurements less than 160 mg/dl, with insulin used only rarely for severe refractory hyperglycemia. Enteral feedings were started between day of life 0 and 2, with maternal colostrum, unfortified pooled pasteurized donor human milk, or preterm formula (24 kcal/oz) per parents’ and treating team’s preference. Maternal milk was typically stored frozen and thawed prior to feeding, although fresh milk was used when available. Feeding volume was ≤5 ml/kg/day on the first day, and increased by no more than 20 ml/kg/day. Minimal target enteral caloric intake was 120 kcal/kg/day, minimal target enteral protein intake was 3.6 g/kg/day, with an optimal target of 4 g/kg/day. Maternal and donor human milk were fortified to ~2-2.3 g/dl protein (24 kcal/oz) with powdered bovine human milk fortifier according to manufacturer directions (4 packets/100 ml) when infants were tolerating at least 25 ml of milk intake per day, with fortification typically occurring when volumes were between 25 and 40 ml per day. We chose to fortify at 25 ml per day at the earliest to avoid wastage of maternal milk, as fortified milk can be kept refrigerated for up to 24 hours before discarding, and one packet of fortifier is mixed in 25 ml of milk. To achieve target protein intake, maternal and donor human milk were fortified further to 2.4-2.65 g/dl protein (27 kcal/oz) in the majority of infants by addition of more human milk fortifier powder (6 packets/100 ml). To achieve target protein intake in infants requiring fluid restriction, milk was fortified by adding 30 ml term formula concentrate and 6 packets HMF/100 ml to provide 2.5-2.7 g/dl protein (30 kcal/oz). Parenteral nutrition was discontinued and central venous lines removed when infants were tolerating 100 ml/kg/day of enteral intake.

### Statistical analysis

Demographic characteristics and clinical outcomes were compared among milk intake groups, using Chi-squared or Fisher’s exact tests for categorical variables, and one-way ANOVA for continuous variables across all study groups. These analyses were repeated for the subgroup of infants receiving > 75% of all in-hospital enteral intake as human milk stratified by human milk type. Growth and nutritional variables were compared in univariate fashion among human milk intake groups and full milk diet subgroups using repeated-measures ANOVA or ANOVA as appropriate. SAS 9.2 (SAS Institute, Cary, NC, USA) was used for all analyses.

## Results

Between January 1, 2003 and June 30, 2005, 224 infants with birthweights less than 1251 g were admitted to UICH NICU by birth or transfer at less than 24 hours’ age. Eighteen infants died before discharge, 32 were transferred to other facilities before 36 weeks’ postmenstrual age, and 3 were excluded for congenital anomalies, leaving 171 subjects included in the analyses. Demographic and clinical characteristics for the all subjects are shown in Table 1. Median postmenstrual-age-specific weight z-score was appropriate for gestational age at birth (−0.4; *i.e.* 35^th^ percentile), and at discharge (−0.94, *i.e.* 17^th^ percentile), but significantly decreased from birth to discharge ( −0.51 change in z-score, p < 0.0001). Infants receiving < 25% human milk (17 days) and those receiving >75% human milk (20 days) had shorter durations of central line and TPN use than those receiving 25-50% human milk (26 days) and those receiving 50-75% human milk (22 days)(p = 0.008, Table 1). 97% of infants received at least some human milk during hospitalization. Only 5 of 171 infants received no human milk and were included in the <25% group. 16% of infants were SGA, (<10^th^ percentile for gestational age) at birth, and 34% of infants were SGA at discharge. Among infants SGA at birth, the majority remained so at discharge (22/27, 81%). Among infants AGA at birth, 21% were SGA at discharge (36/144), with 79% (108/144) remaining AGA.

**Table 1:** Characteristics for all subjects and by human milk intake

		**Group by proportion of enteral intake as human milk**				
	** All subjects **	** <25% **	** 25-50% **	** 50-75% **	** >>75% **	*p* value*
	**n = 171**	**n = 17**	**n = 30**	**n = 36**	**n = 88**	
**Male sex n (%)**	85 (49)	6 (35)	12 (40)	21 (58)	45 (51)	0.52
**Gestational age, weeks, median (IQR)**	27	28.43	26.86	26.64	27	0.63
	(25.4, 28.9)	(25.4, 29.6)	(25.4, 29.0)	(25.7, 28.5)	(25.6, 28.8)	
**Birthweight, g, median (IQR)**	889	1083	861	848	880	0.31
	(724, 1064)	(778, 1184)	(736, 1091)	(717, 1011)	(719, 1052)	
**Birthweight z-score, median (IQR)**	−0.40	−0.32	−0.19	−0.48	−0.45	0.98
	(−0.99, 0.29)	(−0.52, 0.05)	(−0.89, 0.12)	(−1.15, 0.29)	(−0.93, 0.33)	
**SGA at birth n (%)**	27 (16)	3 (18)	4 (13)	7 (19)	13 (15)	0.89
**Maternal antenatal steroids n (%) n = 163**	132 (81)	14 (88)	22 (79)	30 (88)	65 (78)	0.55
**Postnatal dexamethasone n (%)**	53 (31)	5 (29)	10 (30)	12 (34)	25 (28)	0.90
**Age at first enteral feeding, days, median (IQR)**	2 (1, 2)	2 (1, 2)	2 (1, 3)	2 (1, 2)	2 (1, 2)	0.34
**Age at human milk fortification, days, median (IQR) (n = 161)**	14 (9, 22)	10 (6, 25)	19 (8, 25)	13 (10, 20)	14 (9, 21)	0.74
**Central line days, median (IQR)**	22	17	26	22	20	0.008
	(13, 33)	(9, 27)	(15, 37)	(14, 34)	(13, 31)	
**NEC n (%)**	3 (2)	0 (0)	0 (0)	1 (3)	2 (2)	1
**ROP n (%)**	63 (38)	7 (47)	14 (50)	11 (33)	27 (33)	0.32
**PDA n (%)**	85 (49)	5 (29)	16 (53)	17 (49)	45 (51)	0.40
**Sepsis n (%)**	26 (15)	3 (18)	6 (20)	9 (25)	8 (9)	0.09
**IVH n (%)**	31 (18)	1 (6)	4 (13)	2 (6)	23 (27)	0.02
**Oxygen at discharge n (%)**	116 (67)	11 (65)	20 (67)	23 (64)	60 (68)	0.97
**Postmenstrual age at discharge, weeks, median (IQR)**	38.7 (37.1, 40.4)	37.85 (37.1, 40.9)	39.92 (36.9, 41.3)	39.21 (37.42, 40.79)	38.27 (3.07, 39.6)	0.01
**Length of stay, days, median (IQR)**	79 (63, 96)	71 (56, 124)	89 (63, 103)	84 (69, 100)	77 (61, 94)	0.18

* Chi-squared, Fisher’s exact test, or ANOVA across all groups.

### Growth by percent of intake as human milk

There were no significant differences in gestational age, birth weight, birth weight z-score, or gender among groups. Neonatal morbidities were also similar, with no significant difference in incidence of ROP, PDA, NEC, or oxygen at discharge among groups (Table 2). Protein supplementation varied among human milk intake groups. The majority of infants in all groups received protein supplementation beyond the standard human milk fortifier at 2–2.3 g/dl (65-85% of infants), and infants receiving larger proportions of human milk were more likely to receive protein supplementation beyond 2–2.3 g/dl than those receiving less (p = 0.04, Table 2).

**Table 2:** Growth outcomes and human milk fortification for all subjects and by human milk intake

		**Group by proportion of enteral intake as human milk**				** *p* value**	
	**All Subjects**	**<25%**	**25-50%**	**50-75%**	**>75%**	**All groups**	**>75% vs. <75%**
**Discharge weight, g, median (IQR)**	2782 (2460, 3215)	3005 (2470, 3260)	2765 (2500, 3690)	2980 (2695, 3280)	2685 (2335, 3035)	0.008*	0.0006
**Discharge weight z- score, median (IQR)**	−0.94 (−1.43, -0.19)	−0.72 (−1.43, -0.19)	−0.69 (−1.36, -0.05)	−0.95 (−1.28, 0.12)	−1.07 (−1.52, -0.29)	0.36*	0.09
**SGA at discharge n (%)**	58 (34)	7 (41)	8 (27)	9 (25)	34 (39)	0.36*	0.18
**Protein fortification, highest level**							
**n (%)**							
**2.0-2.3 g/dl**	34 (20)	6 (35)	8 (27)	6 (17)	14 (16)	0.04**†**	0.18
**2.4-2.65 g/dl**	84 (49)	6 (35)	14 (46)	16 (44)	48 (55)		
**2.5-2.7 g/dl**	53 (31)	5 (29)	8 (27)	14 (39)	26 (30)		
**Change in weight z-score, birth to discharge, median (IQR)**	−0.51‡ (−0.89, -0.03)	−0.1 (−0.61, 0.16)	−0.30 (−0.85, 0)	−0.32 (−0.73, 0.15)	−0.60 (−0.99, -0.15)	0.17*	0.03

*ANOVA or Chi square across all groups.

**†**Chi square across all fortification levels and all human milk intake groups.

‡ p < 0.0001 – t-test of change in weight z score = 0, infants experienced significant negative change in weight z-score from birth to discharge.

**Figure 1:**
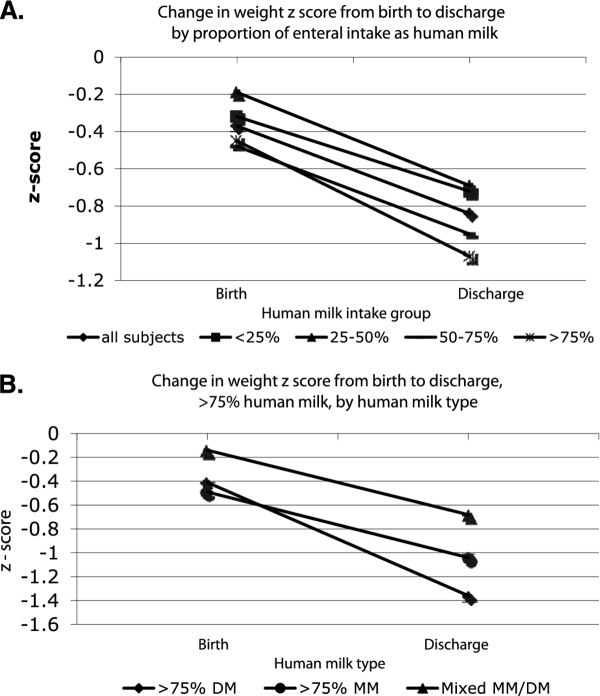
**Change in weight z-score from birth to discharge according to the Fenton growth chart, by proportion of enteral diet as human milk. A**. Change in weight z-score by proportion of enteral intake as human milk, all subjects. **B**. Change in weight z-score by type of human milk diet among infants receiving >75% of enteral intake as human milk. MM = maternal milk; DM = donor human milk; Mixed MM/DM = diet contained >75% human milk, a mixture of both maternal and donor milk, neither of which comprised >75% of the diet.

Change in weight z-score from birth to discharge and discharge weight z-scores did not vary significantly by human milk intake (Table 2), but all groups experienced significant decreases in weight z-score from birth to discharge (p < 0.0001). The decrease in weight z-score was largest for the >75% milk group (−0.6), compared with the other groups (−0.1 to −0.32, p = 0.17) (Figure 1A.). Rates of SGA at discharge did not vary by human milk intake (Table 2). When the > 75% milk group was compared with the rest of the study population as a single group receiving < 75% human milk, infants fed > 75% human milk experienced a significantly larger decrease in weight z-score between birth and discharge (−0.59 vs. -0.36, p 0.03).

### Growth by human milk type

51% of the study population (88/171) received > 75% of all enteral intake during hospitalization as human milk: maternal, donor, or a mixture of both (Table 2). 31 of these 88 infants received > 95% of all enteral intake as human milk, 48 received between 80 and 95%, and 8 received between 75 and 80%. Of the 88 infants who received >75% of intake as human milk, 23 received >75% donor milk, 51 received >75% maternal milk, and the remaining 14 received a combination of both types of milk with neither comprising 75% of the diet (Table 3). 17 infants in the donor milk group received maternal milk as well, and 16 infants in the maternal milk group received donor milk. There were no significant differences in birthweight, birthweight z score, gestational age, or sex among human milk type subgroups. There were no statistically significant differences in the prevalence of neonatal morbidities that may influence growth, including NEC, PDA, sepsis, ROP, IVH, oxygen at discharge, nor in the use of postnatal dexamethasone to treat BPD between milk type subgroups (data not shown).

**Table 3:** Growth outcomes and human milk fortification, >75% human milk diet group

	**Subgroup by type of human milk**			** *p* value all 3 groups**	** *p* value > 75% DM vs. >75% MM**
	**>75% DMn = 23**	**>75% MMn = 51**	**Mixed MM/DMn = 14**		
**Birthweight, g, median (IQR)**	868	993	769		
	(643, 997)	(767, 1100)	(694, 822)	0.07	0.22
**Birthweight z-score, median, (IQR)**	−0.41	−0.49	−0.14		
	(−0.99, -0.03)	(−0.90, 0.48)	(−0.66, 0.35)	0.74	0.66
**Discharge weight, g median (IQR)**	2330	2710	2875		
	(2070, 2720)	(2480, 3050)	(2500, 3200)	0.07	0.09
**Discharge weight z-score median (IQR)**	−1.36	−1.04	−0.68		
	(−1.83, -0.48)	(−1.43, -0.26)	(−1.2, -0.17)	0.24	0.16
**SGA at discharge n (%)**	13 (56)	18 (35)	3 (21)	0.08	0.13
**Protein fortification, highest level used n (%)**					
**2.0-2.3 g/dl**	3 (13)	10 (20)	1 (7)		
**2.4-2.65 g/dl**	13 (57)	27 (53)	8 (57)		
**2.5-2.7 g/dl**	7 (30)	14 (27)	5 (36)	0.82	1
**Discharge head circumference, cm**	32	33.5	33.25	0.23	0.10
**(n = 56) median (IQR)**	(31.5, 33.5)	(32.5, 34.75)	(32.25, 34.25)		
**Discharge head**	−0.7	−0.4	−0.9		
**circumference z-score median (IQR)**	(−1.4, -0.2)	(−1, 0.4)	(−1.15, -0.25)	0.11	0.10
**Change in weight z-score, birth to discharge median (IQR)**	−0.84 (−1.09, -0.25)	−0.56 (−0.89, -0.03)	−0.45 (−1.20, -0.15)	0.54	0.28

The majority of all infants receiving >75% of enteral intake as human milk received protein and calorie supplementation beyond standard 24 kcal/oz (84-93%, Table 3), and the pattern of protein and calorie supplementation did not vary significantly by milk type.

Change in weight z-score from birth to discharge, discharge weight z-score, and discharge OFC z-score did not differ significantly by human milk type, both when all three milk type groups were compared, and when donor milk and maternal milk were directly compared (Table 3)(Figure 1B). There was a trend toward higher rates of SGA at discharge in donor milk fed infants (56%) than in those fed maternal milk (35%) or mixed donor and maternal milk (21%)(p = 0.08, Table 3).

## Discussion

Our population of VLBW infants fed predominantly human milk experienced low rates of SGA at discharge, and average loss of one-half of a standard deviation in weight for gestational age between birth and discharge. Although increasing proportions of enteral intake as human milk were not associated with increasing detriment in weight z-score, infants fed >75% human milk experienced significantly larger decrease in weight z-score from birth to discharge when compared with infants fed < 75% (p = 0.03). In addition, among infants fed > 75% human milk, infants fed predominantly donor human milk demonstrated higher rates of SGA at discharge than those fed maternal milk or mixed donor and maternal milk (p = 0.08).

The study infants grew well overall by the definition of being AGA at birth (z-score −0.4, *i.e.*35^th^ percentile), and appropriate for postmenstrual age at discharge (z-score −0.94, *i.e.* 17^th^ percentile). In-hospital growth failure, as defined by SGA status at 36 weeks’ postmenstrual age, has previously been described as a problem in the VLBW population. Dusick and colleagues described a population of 1433 infants from the NICHD Neonatal Research Network (NRN) born between 2000 and 2001. They determined that while only 16% of VLBW infants were SGA at birth, 89% met this definition at 36 weeks postmenstrual age [14]. Those outcomes represented an apparent improvement in rates of growth failure among VLBW infants in the NRN, as 97% of 4438 infants admitted to NRN centers between 1995 and 1996 were SGA at postmenstrual age at 36 weeks [20]. In contrast, 34% of our subjects were SGA at discharge, which occurred at a median postmenstrual age of 38.7 weeks, and only 21% of infants who were AGA at birth had become SGA by the time of discharge.

As reported by other investigators, predominantly human milk diets in our population (>75%) resulted in significantly slower growth, as defined by a statistically significantly larger decrease in weight z-score between birth and discharge, than diets containing <75% human milk, although no dose–response effect was noted between increasing proportion of human milk and growth. Schanler and colleagues studied a similar population of VLBW infants, comparing those fed an average of 84% of the in-hospital diet as fortified human milk to those fed solely preterm formula. They found that human-milk-fed infants experienced slower weight gain, and were 500 g lighter at discharge than their formula-fed peers [2]. Using the Fenton chart to calculate z-scores for Schanler’s study results in a weight z-score change of −1.86 for the human milk group compared with a weight z-score change of −1.34 for the formula group. O’Connor and colleagues also found a negative dose–response relationship between human milk intake and in-hospital growth as measured by weight at term-adjusted age. Among their population of larger VLBW infants (mean GA 30 wks, 1275 g birthweight), infants fed > 80% fortified human milk weighed 500 g less at term-adjusted age than those fed solely formula [11]. Negative change in weight z-score calculated using the Fenton chart from birth to term in their study was also related to human milk, with the largest decline seen among those fed the most human milk (−1.62), and the least decline seen with exclusive formula feeding (−0.64).

A predominantly donor milk diet was associated with higher rates of growth failure as defined by discharge weight < 10^th^ percentile for postmenstrual age (SGA) in our population, compared with predominantly maternal milk and mixed human milk diets (p = 0.08). Infants fed predominantly mixed human milk diets had the lowest rates of SGA of all infants in the subgroup of infants fed >75% human milk. Although all infants included in the >75% human milk subgroup analysis received >75% human milk, intake varied systematically. Infants in the mixed diet group received 75-80% human milk, while infants in the maternal and donor groups received 85-100% human milk. The rate of SGA in the mixed group (21%) is therefore closer to the rate of SGA for the 50-75% human milk group (25%) than that of the >75% human milk group considered as a whole (39%) (Tables 2 and 3). Sullivan and colleagues also reported growth outcomes in a population of VLBW infants fed >70% maternal milk, some of whom received a formula supplement as the remainder of the diet, and some of whom received donor human milk [3]. Infants receiving donor human milk in this trial also received human milk human milk fortifier, in contrast to the bovine milk human milk fortifiers used both in all other studies discussed and in our subjects. Infants fed the donor milk supplement showed a trend toward slower weight gain than those fed a formula supplement (p = 0.13).

A possible explanation for slower growth rates among infants fed fortified human milk, in general, and donor milk, specifically, is the inadequate protein content of standard fortified human milk for the needs of VLBW infants. Growth of preterm infants is linearly related to protein intake [21]. Enteral protein requirements to achieve in-utero growth rates in VLBW infants range between 4 g/kg/day for the <1000 g infants and 3.6 g/kg/day for 1200–1500 g infants [22]. Despite the higher protein content of milk produced by mothers delivering preterm [23,24] and routine use of human milk fortifiers, VLBW infants fed human milk experience a protein deficit during the initial months of life. This deficit has been estimated by Arsanoglu et al, who compared assumed protein intake for VLBW infants to actual intake, to be 0.5 to 0.8 g/kg/day [25].

Inadequate protein intake is even more pronounced when donor milk is considered. Most donor human milk is obtained from donors who delivered term infants and have been lactating for several months, and thus does not contain the higher preterm protein levels. There is evidence that donor milk contains even lower protein content than standard mature human milk, possibly due to processing and handling. Wojcik et al studied 415 samples of unpooled donor milk from 273 donors and found that the median protein content was 1 g/dl and that 30% of samples contained < 1 g/dl [26]. Mean protein content in 39 samples from donors to the Mother’s Milk Bank of Ohio was 0.9 mg/dl [27]. Using standard powdered bovine HMF with donor milk thus results in a diet severely deficient in protein, i.e. 2.8-3 g/kg/day, increasing the risk for growth failure.

Growth can be optimized with human milk in general, and donor milk specifically, if this relative protein deficiency is recognized and diets are modified to provide adequate protein intake. Protein intake can be optimized in either individualized or group methods [28,29]. The approach now taken in our unit is to increase protein intake for all infants, with target intake of 3.6-4 g/kg/day, without attempting to individualize intake. Our findings of lower rates of growth failure than other investigators is likely attributable to higher protein intake in our predominantly human-milk-fed subgroup of VLBW infants. However, as multi-component fortifiers were used to achieve target protein intakes (bovine human milk fortifiers and term formula concentrate) in our population, overall caloric content of the milk was also increased in addition to protein. Some of the growth results obtained with our approach may be due simply to higher caloric density of the milk, however, protein intake has been found to be strongly linked to growth in VLBW infants [21].

This study’s strengths include the prospectively collected growth and nutritional information and the use of z-scores, which allow for comparison of growth outcomes to a recognized standard. Our study also includes a significant number of VLBW infants fed donor human milk, and allows us to compare predominantly donor milk to predominantly maternal milk diets.

Our study’s weaknesses include the limited sample size and observational nature. Using an observational study design did not allow us to control enteral intake or compare different protein intakes in a randomized fashion. Use of donor milk or specific protein fortification schemes may have been systematically associated with other infant factors that impacted growth, creating residual confounding. In addition, we collected weight data only at birth and discharge, and were not able to assess the timing of the lowest weight z-score experienced by the infants, or how the trajectory of weight growth may have changed from day to day or week to week during hospitalization. We do not assume that the weight z-score at discharge represents the lowest weight z-score, we only report the change between birth and discharge. We were also unable to assess the effects of donor or maternal human milk on linear growth in our population, as standardized length data were not obtained. Ramel and colleagues have recently demonstrated that poor in-hospital linear growth is associated with poor cognitive outcomes in former VLBW infants at age 2, and that length z-scores for gestational age were lower than weight z-scores in their population [30]. Future studies should include standardized length measurements. Another limitation of our study is that we did not measure caloric intake or protein intake directly, and therefore we are unable to report the intakes needed to achieve the growth results we demonstrated.

## Conclusions

Human milk offers many benefits for VLBW infants, and should be the default diet for all such infants. We demonstrate that in-hospital growth of VLBW infants can be adequate with predominant human milk diets, both maternal and donor derived. Our population experienced low rates of SGA at discharge, and average loss of one-half of a standard deviation in weight for gestational age between birth and discharge. Donor human milk diets may be associated with more growth challenges than maternal milk diets. Special attention to protein and calorie fortification is necessary to provide the benefits of a human milk diet without sacrificing growth.

## Competing interests

The authors declare that they have no competing interests.

## Authors’ contributions

TTC designed the study, performed the statistical analysis, and drafted the manuscript. SC collected the nutritional and clinical information and participated in study design. AFS aided in analysis design and in the manuscript editing. FHM aided in study design and aided in drafting and editing the manuscript. All authors read and approved the final manuscript.

## Funding

NIH K23HD057232 provided funding for this study by supporting the salary of TTC. The NIH had no part in the study design, collection, analysis, or interpretation of data, or in the decision to submit this manuscript for publication.

## Pre-publication history

The pre-publication history for this paper can be accessed here:

http://www.biomedcentral.com/1471-2431/12/124/prepub

## References

[B1] Schanler RJ Lau C Hurst NM Smith EOB Randomized trial of donor human milk versus preterm formula as substitutes for mothers' own milk in the feeding of extremely premature infants Pediatrics 2005 116 2 400 406 10.1542/peds.2004-1974 16061595

[B2] Schanler RJ Shulman RJ Lau C Feeding strategies for premature infants: beneficial outcomes of feeding fortified human milk versus preterm formula Pediatrics 1999 103 6 Pt 1 1150 1157 10353922 10.1542/peds.103.6.1150

[B3] Sullivan S Schanler RJ Kim JH Patel AL Trawoger R Kiechl-Kohlendorfer U Chan GM Blanco CL Abrams S Cotten CM An Exclusively Human Milk-Based Diet Is Associated with a Lower Rate of Necrotizing Enterocolitis than a Diet of Human Milk and Bovine Milk-Based Products J Pediatr 2010 156 4 562 7e1 10.1016/j.jpeds.2009.10.040 20036378

[B4] Meinzen-Derr J Poindexter B Wrage L Morrow AL Stoll B Donovan EF Role of human milk in extremely low birth weight infants' risk of necrotizing enterocolitis or death J Perinatol 2009 29 1 57 56 10.1038/jp.2008.117 18716628 PMC2801431

[B5] el-Mohandes AE Picard MB Simmens SJ Keiser JF Use of human milk in the intensive care nursery decreases the incidence of nosocomial sepsis J Perinatol 1997 17 2 130 134 9134512

[B6] Hylander MA Strobino DM Dhanireddy R Human milk feedings and infection among very low birth weight infants Pediatrics 1998 102 3 E38 10.1542/peds.102.3.e38 9724686

[B7] Lucas A Morley R Cole TJ Lister G Leeson-Payne C Breast milk and subsequent intelligence quotient in children born preterm.[see comment] Lancet 1992 339 8788 261 264 10.1016/0140-6736(92)91329-7 1346280

[B8] Vohr BR Poindexter BB Dusick AM McKinley LT Higgins RD Langer JC Poole WK Persistent beneficial effects of breast milk ingested in the neonatal intensive care unit on outcomes of extremely low birth weight infants at 30 months of age Pediatrics 2007 120 4 e953 e959 10.1542/peds.2006-3227 17908750

[B9] Morley R Lucas A Randomized diet in the neonatal period and growth performance until 7.5–8 y of age in preterm children Am J Clin Nutr 2000 71 3 822 828 10702179 10.1093/ajcn/71.3.822

[B10] Morley R Cole TJ Powell R Lucas A Mother's choice to provide breast milk and developmental outcome Arch Dis Child 1988 63 11 1382 1385 10.1136/adc.63.11.1382 3202647 PMC1779171

[B11] O'Connor DL Jacobs J Hall R Adamkin D Auestad N Castillo M Connor WE Connor SL Fitzgerald K Groh-Wargo S Growth and development of premature infants fed predominantly human milk, predominantly premature infant formula, or a combination of human milk and premature formula J Pediatr Gastroenterol Nutr 2003 37 4 437 446 10.1097/00005176-200310000-00008 14508214

[B12] Ehrenkranz RA Younes N Lemons JA Fanaroff AA Donovan EF Wright LL Katsikiotis V Tyson JE Oh W Shankaran S Longitudinal growth of hospitalized very low birth weight infants Pediatrics 1999 104 2 Pt 1 280 289 10429008 10.1542/peds.104.2.280

[B13] Lucas A Gore SM Cole TJ Bamford MF Dossetor JF Barr I Dicarlo L Cork S Lucas PJ Multicentre trial on feeding low birthweight infants: effects of diet on early growth Arch Dis Child 1984 59 8 722 730 10.1136/adc.59.8.722 6476868 PMC1628628

[B14] Dusick AM Poindexter BB Ehrenkranz RA Lemons JA Growth failure in the preterm infant: can we catch up? Semin Perinatol 2003 27 4 302 310 10.1016/S0146-0005(03)00044-2 14510321

[B15] Ehrenkranz RA Dusick AM Vohr BR Wright LL Wrage LA Poole WK Growth in the neonatal intensive care unit influences neurodevelopmental and growth outcomes of extremely low birth weight infants Pediatrics 2006 117 4 1253 1261 10.1542/peds.2005-1368 16585322

[B16] HMBANA - Who do we serve? http://www.hmbana.org/who-do-we-serve

[B17] Stein H Cohen D Herman AA Rissik J Ellis U Bolton K Pettifor J MacDougall L Pooled pasteurized breast milk and untreated own mother's milk in the feeding of very low birth weight babies: a randomized controlled trial J Pediatr Gastroenterol Nutr 1986 5 2 242 247 3514832

[B18] Tyson JE Lasky RE Mize CE Richards CJ Blair-Smith N Whyte R Beer AE Growth, metabolic response, and development in very-low-birth-weight infants fed banked human milk or enriched formula. I. Neonatal findings Journal of Pediatrics 1983 103 1 95 104 10.1016/S0022-3476(83)80790-2 6864403

[B19] Fenton TR A new growth chart for preterm babies: Babson and Benda's chart updated with recent data and a new format BMC Pediatr 2003 3 13 10.1186/1471-2431-3-13 14678563 PMC324406

[B20] Lemons JA Bauer CR Oh W Korones SB Papile LA Stoll BJ Verter J Temprosa M Wright LL Ehrenkranz RA Very low birth weight outcomes of the National Institute of Child health and human development neonatal research network, January 1995 through December 1996. NICHD Neonatal Research Network Pediatrics 2001 107 1 E1 10.1542/peds.107.1.e1 11134465

[B21] Kashyap S Schulze KF Forsyth M Dell RB Ramakrishnan R Heird WC Growth, nutrient retention, and metabolic response of low-birth-weight infants fed supplemented and unsupplemented preterm human milk Am J Clin Nutr 1990 52 2 254 262 2375291 10.1093/ajcn/52.2.254

[B22] Ziegler EE Protein requirements of very low birth weight infants J Pediatr Gastroenterol Nutr 2007 45 Suppl 3 S170 S174 10.1097/01.mpg.0000302966.75620.91 18185086

[B23] Anderson DM Williams FH Merkatz RB Schulman PK Kerr DS Pittard WB Length of gestation and nutritional composition of human milk Am J Clin Nutr 1983 37 5 810 814 6846220 10.1093/ajcn/37.5.810

[B24] Neville MC McManaman JL Thureen PJ, Hay WW Milk secretion and composition Neonatal Nutrition and Metabolism. edn 2006 New York: Cambridge University Press 377 389

[B25] Arslanoglu S Moro GE Ziegler EE Preterm infants fed fortified human milk receive less protein than they need J Perinatol 2009 29 7 489 492 10.1038/jp.2009.50 19444237

[B26] Wojcik KY Rechtman DJ Lee ML Montoya A Medo ET Macronutrient analysis of a nationwide sample of donor breast milk J Am Diet Assoc 2009 109 1 137 140 10.1016/j.jada.2008.10.008 19103335

[B27] Valentine CJ Morrow G Fernandez S Gulati P Bartholomew D Long D Welty SE Morrow AL Rogers LK Docosahexaenoic acid and amino acid contents in pasteurized donor milk are low for preterm infants J Pediatr 2010 157 6 906 910 10.1016/j.jpeds.2010.06.017 20850762

[B28] Arslanoglu S Moro GE Ziegler EE Adjustable fortification of human milk fed to preterm infants: does it make a difference? J Perinatol 2006 26 10 614 621 10.1038/sj.jp.7211571 16885989

[B29] Polberger S Raiha NC Juvonen P Moro GE Minoli I Warm A Individualized protein fortification of human milk for preterm infants: comparison of ultrafiltrated human milk protein and a bovine whey fortifier J Pediatr Gastroenterol Nutr 1999 29 3 332 338 10.1097/00005176-199909000-00017 10468001

[B30] Ramel SE Demerath EW Gray HL Younge N Boys C Georgieff MK The relationship of poor linear growth velocity with neonatal illness and two-year neurodevelopment in preterm infants Neonatology 2012 102 1 19 24 10.1159/000336127 22441508

